# Risk of psychosis in refugees: a literature review

**DOI:** 10.1038/tp.2017.119

**Published:** 2017-06-13

**Authors:** J Dapunt, U Kluge, A Heinz

**Affiliations:** 1 Department of Psychiatry and Psychotherapy, Charité – Universitätsmedizin Berlin, Berlin, Germany

## Abstract

Conflicts and precarious living conditions resulted in the arrival of large numbers of refugees in Europe and especially in Germany. Evidence suggests that immigrant populations are at elevated risk of psychotic disorders. Considering the traumatic pre- and post-migratory adversities refugees may have encountered, people granted refugee status may even be more susceptible to psychosis than non-refugee migrants. The aim of this literature review is to summarise and interpret recent research on the incidence or prevalence of psychotic disorders in refugees, additionally focusing on the aspects of gender and Middle Eastern provenance. A systematic search in PubMed was performed in the time from 20 to 28 May 2016. Relevant literature was limited to articles describing cohort studies conducted in Western industrialised countries. Articles published between 1 June 2006 and 28 May 2016 were analysed. Content relating to psychotic disorders in refugees was reviewed and summarised. The selected studies showed an increased risk of psychotic disorders in refugees compared with both the indigenous population and non-refugee. migrants. The elevated risk was more pronounced in refugee men. A particularly high risk in refugees of Middle Eastern origin could not be inferred. The higher susceptibility to psychotic disorders in refugees emphasises the need for the development and implementation of adequate prevention strategies. Clinicians and people working in a refugee setting should be aware of early signs and symptoms of psychosis. Further research is required to evaluate post-migratory experiences and investigate the population of refugees affected by the current humanitarian crisis.

## Introduction

### Definitions

In the context of this literature review, the following differences between the concerned population categories will be pointed out.

A refugee is ‘a person who has fled their own country because they have suffered human rights abuses or because of who they are or what they believe in’^1^ (the issue in detail, para. 3).

In contrast, ‘an asylum-seeker is someone who has left their country in search of international protection, but is yet to be recognised as a refugee’^1^ (the issue in detail, para. 4).

Refugees and asylum seekers are migrants, but there are also other subgroups, like regular migrants, who have a visa or a legal residence status. The distinction between refugees and migrants is bound on the ability of the latter to choose to move, they usually do not face potentially deadly consequences as a result of asylum denial.^2^

### The refugee crisis

The number of refugees is constantly rising, in Germany, in Europe and worldwide. By the end of 2014, some 19.5 million people were refugees and an estimated 1.8 million people were asylum-seekers. During 2014, an average of 42 500 of individuals per day were forced to flee their home as a result of persecution, human rights abuses, violence or precarious living conditions, resulting in 2.9 million new refugees. This compares to 32 200 per day in 2013 and 10 900 in 2010. Fifty-three percent of all refugees worldwide came from just three countries: Syria, Afghanistan and Somalia, accounting for 3.88 million, 2.59 million and 1.11 million people, respectively. A record high of nearly 1.7 million applications for asylum or refugee status was reported in 2014.^3^

In 2015, Germany recorded the highest level of immigration since 1950, registering a net immigration gain of 1 139 000 people.^4^ Eight percent of all immigrants entered Germany on humanitarian grounds, the main reason being the acceptance of Syrian citizens and the residence permissions conceded to asylum seekers (24%).^4, 5^ Syria was in fact the predominant country of origin for refugees comprising 15% of all immigrants and 26% of all asylum seekers between 2011 and 2015.^4^ In 2015, 35.9% of all asylum seekers were Syrian citizens.^5^ In 2015, 476 649 applications for asylum were filed, that is, an increase of 135% compared with 2014.^4, 5^ This makes Germany the top receiving country among industrialised nations. Another 56% increase in applications for asylum was registered in 2016 compared with 2015, mainly due to delayed applications. In total, 321 371 asylum seekers were registered in Germany in 2016.^6^

### Migration and psychosis

Meta-analytic reviews^7, 8^ found a two- to threefold increased risk of psychosis in migrants compared with the host population. The outcomes suggest that the risk varies by country of origin and host country and is even higher for second-generation migrants. Typically, high incidence rates are not found in the country of origin, suggesting that the experiences preceding and following the migration process may play a role in the aetiology of psychotic disorders.^9^ People granted refugee status may therefore be particularly vulnerable to psychosis, considering their exposure to violence, conflict, persecution, discrimination and other forms of psychosocial disadvantage, such as material strains, persistent lack of prospects, scarce possibilities to partake in society and, accompanied therewith, social isolation. A susceptibility to develop symptoms of dissociative nature may as well be expected in this population, as childhood adversity has been associated with the presentation of dissociative symptoms.^10^ Notably, childhood abuse, neglect and paternal problems have been identified as important predictors of dissociation.^11, 12, 13^ In view of the likelihood for refugees of having suffered such afflictions, a vulnerability to develop dissociative symptoms in addition to an increased risk for psychosis may be found in this population.

### Aims

The aim of this literature review is to identify and analyse studies conducted in the last 10 years that investigate the incidence of psychotic disorders in refugees. More precisely, diagnoses according to the ICD-10 criteria F20–29 (schizophrenia, schizotypal and delusional disorders) as well as the respective criteria stated in the DSM-5 manual (schizophrenia spectrum and other psychotic disorders) are taken into account. This review will focus on the incidence rates found among refugees compared with other migrants or the host country’s native population, on differences between genders and on their region of origin. In addition to the review and summary of the main results, a comparison with the findings of previously published meta-analyses and literature reviews will be given. In conclusion, an outline on possible prevention strategies and further research will be discussed.

## Methods

This assignment was written in line with the declarations stated in the statute for the assurance of good scientific practice (Satzung zur Sicherung guter wissenschaftlicher Praxis) of the Charité – Universitätsmedizin Berlin. An approval by the Ethical Review Committee was not present.

### Inclusion and exclusion criteria

All articles describing original research investigating the incidence or prevalence of psychosis in refugees, compared with either the native population and/or migrants with non-refugee status were included in the review. The diagnosis of schizophrenia, schizotypal and delusional disorders according to the ICD-10 classification had to be made (F20–29) (or equivalent diagnosis according to the DSM-5 classification). Publications in English, German, Italian and French were considered. In order to allow comparison between the studies and discussion of the result’s transfer to Germany, only studies conducted in Western industrialised countries were included.

Studies that did not diagnose a psychotic disorder classified as F20–29 in the ICD-10 system (or equivalent DSM-5 diagnosis) were discounted, as were the articles that did not differentiate between migrants granted refugee status or asylum seekers and non-refugee migrants.

### Search strategy

A systematic query was performed for published articles describing original research. Database research using the search terms psychosis AND refugee* was performed in MEDLINE (PubMed) in the time from 20 to 28 May 2016 and, for the purpose of completeness, in Psychological Index (PSYNDEX) in the time from 28 January to 1 February 2017. In order to ensure the inclusion of articles containing slightly different terms or synonyms thereof, a MeSH (Medical Subject Heading) term search was carried out using the search details shown below.

(‘psychotic disorders’[MeSH Terms] OR (‘psychotic’[All Fields] AND ‘disorders’[All Fields]) OR ‘psychotic disorders’[All Fields] OR ‘psychosis’[All Fields]) AND (refugee[All Fields] OR refugee'[All Fields] OR refugee's[All Fields] OR refugeehood[All Fields] OR refugeeism[All Fields] OR refugeeness[All Fields] OR refugees[All Fields] OR refugees'[All Fields] OR refugeetherapy[All Fields]).

The publication date was limited from 1 June 2006 to 28 May 2016. According to the inclusion and exclusion criteria, relevant articles were identified by inspecting the abstract and, where needed, the full text of the article.

### Data extraction

All studies were assessed with regard to population size, characteristics of the control group, mean age of both the refugee and control population, distribution of gender, diagnostic criteria, follow-up time and statistical analysis by means of the National Institutes of Health’s quality assessment tool for cohort studies.^14^

## Results

### Search results

**Figure 1:**
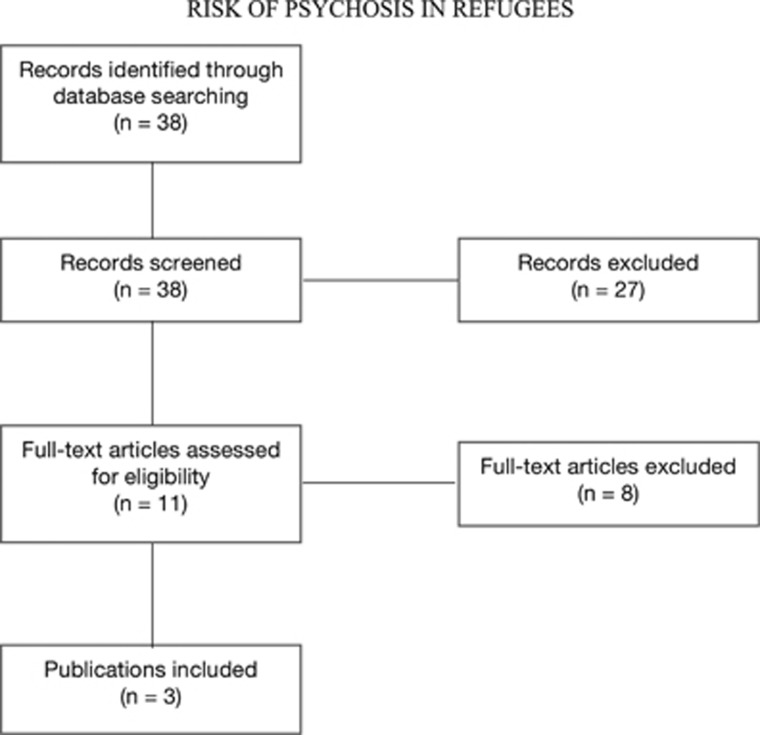
Schematic representation of study selection.

The query in MEDLINE yielded 38 results. After inspecting the titles and the abstract of the publications, and, where needed, the full text, three articles met the inclusion criteria. Articles were excluded for the following reasons: non-relevant subject (*n*=9), case report (*n*=1), commentary or editorial (*n*=3), literature review or meta-analysis (*n*=7), full text not available or language other than English, German, Italian or French (*n*=4), not meeting the inclusion criteria (*n*=11). Content in the three relevant articles relating to the diagnosis of psychosis in refugees was identified, analysed and interpreted. An overview of the selection process is shown in Figure 1.

Concerning the query in PSYNDEX, we found 1471 articles. None of them met the aforementioned inclusion criteria.

### Characteristics of the studies

All selected articles described retrospective cohort studies. The main characteristics of the studies are presented in Table 1.

The findings of all three studies^15, 16, 17^ are strengthened by the use of large databases or national registers. The reference groups were matched to the cohort according to age and gender, thus guaranteeing accuracy and representativeness of the outcomes. In all studies,^15, 16, 17^ adjustment for age and gender was calculated for the purpose of data analysis, yet only Hollander *et al.* and Anderson *et al.*^15, 16^ adjusted their data with regard to income and population density (or urban residence in the case of Anderson *et al.*^16^). Norredam *et al.*^17^ analysed their data regardless of those factors. Although second-generation migrants were not included in two studies,^15, 17^ they were misclassified as part of the reference group by Anderson *et al.*^16^ Anderson *et al.*^16^ also included any people who originally landed in a Canadian province other than Ontario in the reference group.

### Results of the studies

The main findings of the studies regarding the incidence and hazard ratios for refugees and the control groups as well as gender and Middle Eastern origin are reported and combined in Table 2.

#### Refugees compared to native population

Compared with the local population, there was evidence of higher rates of psychotic disorders in people granted refugee status in all selected studies.^15, 16, 17^ Two studies^15, 16^ found greater incidence rates among the refugee group compared to the indigenous population: Hollander *et al.*^15^ observed a crude incidence rate of non-affective psychotic disorders of 126.4 (95% confidence interval 103.1–154.8) per 100 000 person years in refugees as opposed to 38.5 (95% CI 37.2–39.9) per 100 000 person years in the Swedish-born population, whereas the incidence rate of psychotic disorders identified by Anderson *et al.*^16^ was at 72.8 (95% CI 67.1–78.9) per 100 000 person years among refugees and at 55.6 (95% CI 54.9–56.4) among the general population. One study^17^ scrutinised first-time psychiatric contacts revealing prevalence percentages of 7.3% in refugees (*n*=29 139) and 4.3% in the control group (*n*=116 556). Using national statistical registers, cohorts were selected according to their residence permission (as refugees or through family reunification) and had to be at least 18 years of age upon obtainment. The control group consisted of native Danes born to Danish-born parents, thus excluding second-generation migrants, and were matched to the refugee group in terms of gender and age. Regarding psychotic disorders, refugees had a twofold increased risk of having a first-time psychiatric contact in comparison with native Danes (rate ratio=2.03; 95% CI 1.72–2.40). Considering first-time psychiatric contacts, elevated rate ratios were found in refugees of both sexes.^17^ Using the Swedish-born population as the reference group, Hollander *et al.*^15^ calculated hazard ratios of 3.61 (95% CI 2.87–4.53) and 2.90 (95% CI 2.31–3.64) for refugees.

#### Refugees compared to non-refugee migrants

Two studies^15, 16^ also used non-refugee migrants as a reference group. Hollander *et al.*^15^ observed a higher incidence rate per 100 000 person years in refugees compared to other migrants as well as an approximately two-fold risk to develop a non-affective psychosis compared with non-refugee migrants (hazard ratio=1.58, 95% CI 1.26–1.99; 1.66, 95% CI 1.32–2.09, for more detail see Table 2). As a lower incidence rate per 100 000 person years was found for psychotic disorders among migrants in Canada compared with the general population, the incidence rate ratio estimated (1.24, 95% CI 0.86–1.81) indicated a marginally higher risk for refugees but was statistically not significant compared to the general population. However, refugee status was determined as a predictor of an elevated risk of a psychotic disorder (incidence rate ratio=1.27, 95% CI 1.04–1.56) with non-refugees as the reference group.^16^

### Gender

All studies showed that refugee men had a higher risk of both having a first-time psychiatric contact^17^ and being diagnosed with a psychotic disorder than did refugee women (for more details see Table 2).^15, 16, 17^ This finding was consistent with all reference groups, that is, comparison with the native population, migrants or non-refugee migrants from the same region of origin.^15, 16, 17^ The outcomes were most striking for refugee men in Sweden, for whom the hazard ratio indicated a four-fold increased risk compared with the indigenous population (hazard ratio=4.28, 95% CI 3.28–5.58; women: 2.65, 1.80–3.92) after adjustment for age at risk, gender and their interaction.^15^ Albeit statistically not significant, the analysis of the effect of gender in Denmark^17^ reported a lower risk of having a first-time psychiatric contact for refugee women (RR=0.88, 95% CI 0.74–1.05) in comparison to refugee men. Conversely, the risk was significantly higher in native Danish women (RR=1.20, 95% CI 1.07–1.34) than in Danish-born men.^17^ In addition, the observations made by Norredam *et al.*^17^ concerning first-time contact for mental disorders revealed significant differences between male and female refugees born in Middle Eastern countries (including North Africa) (rate ratio for men: 2.76, 95% CI 2.24–3.41; women 1.60, 1.18–2.19) and was even more pronounced in Iraqi refugees (rate ratio for men: 3.31, 95% CI 2.85–3.83; women 1.95, 1.47–2.59).

### Middle Eastern origin

All studies^15, 16, 17^ found evidence that the rate of psychosis varied by region of origin (*P*=0.05 in Hollander *et al.*^15^). Refugees originating from the Middle East region and North Africa were found to be at elevated risk for psychotic disorders compared with both the Danish-born participants and the refugee group as a whole (RR=3.10, 95% CI 2.28–4.21).^17^ Refugees of Middle Eastern origin were at the highest risk among all screened countries of origin, except Eastern Europe.^17^ Iraqi and Middle Eastern refugees also had a significantly increased risk of having a first-time contact for mental disorder compared with native Danes.^17^ Given the high number of Iraqi citizens, Norredam *et al.*^17^ differentiated between refugees with Iraqi citizenship and other Middle Eastern countries. Refugees of Iraqi origin had higher rate ratios for first-time psychiatric contact compared with other Middle Eastern countries.^17^ Nonetheless, the inverse effect was encountered analysing the participants diagnosed with psychosis, for which refugees coming from the Middle East region were at higher risk than did Iraqi refugees.^17^ Hollander *et al.*^15^ determined a greater incidence rate in refugees from the Middle East region (including North Africa) compared with both the Swedish-born and migrants from the Middle East region with non-refugee status. In Canada, only slight differences were found in refugees coming from West Central Asia and the Middle East compared with the general population and all refugees.^16^

## Discussion

### Principal results

In summary, refugees were found to have an increased risk for psychotic disorders in comparison with both the indigenous population and non-refugee migrants. A particularly high risk was detected in male refugees. The findings pertaining to the Middle East as the region of origin were not concordant. Hence, an elevated risk for this subpopulation of refugees compared with refugees of different origin could not be confirmed. Nevertheless, it is important to point out that considerable differences between the various regions of provenance were observed in all three citations.^15, 16, 17^

### Comparison with other literature

The outcomes tally with a wide range of literature that reviewed the effect of migration on the aetiology of schizophrenia.^7, 8, 18, 19^ The findings of the Canadian study^16^ that describe no elevated risk in non-refugee migrants are nonetheless contrary to the previous meta-analytic reviews,^7, 8, 19^ thus complicating their interpretation in light of an ethnically diverse Canadian baseline population. The increased risk in refugees reported by Norredam *et al.*^17^ was not compared to that of other migrants. Thus, attribution to a direct refugee effect is limited.

Altogether, the principal results compare favourably with those reported in other literature:

A meta-analytic review scrutinising the rates of psychosis in England dating from 1950 to 2009^20^ encountered elevated rates for most disorders in several ethnic minority groups compared with the ‘white’ British population. In spite of the small number of patients analysed (*n*=104), a descriptive investigation concerning refugees and asylum seekers in London^21^ found that psychotic disorders were the most common diagnosis (53%). Similar findings were reported in a pilot study conducted in 12 psychiatric hospitals in Germany:^22^ schizophrenia was the most common diagnosis (36.1%) in migrants (including refugees, non-refugees and second-generation migrants). Those of Arab origins were at a particular high risk. The authors also claimed that asylum seekers and refugees comprised a large percentage of the migrants in treatment (9.23%) and that long-term treatment was more frequent in migrants than in the indigenous population. A literature review by Parrett and Mason^23^ presents evidence that either the prevalence or incidence of psychotic disorders is consistently higher in refugee populations compared to non-refugees. This outcome is corroborated by a large size of findings both from small sample studies and larger population-based studies, which all show greatly increased risk. Accordingly, these results support the hypothesis contending that psychosis can be a consequence of forced migration.^23^

### Limitations

This literature review investigated whether refugees are at elevated risk for developing a non-affective psychosis. The initial assumption was confirmed, as all studies^15, 16, 17^ contended that refugees are indeed more susceptible to psychotic disorders. There are, however, some inherent limitations that have some bearing on the validity of those findings. The central shortcoming of this review is the small number of articles included. The analysis of a larger body of literature, using multiple databases for research, would therefore be needed to strengthen the validity of the outcomes. Furthermore, representativeness is also limited because the ethnical composition of refugees along with psychosocial and political implications differ widely between countries. Thus, extrapolating conclusions should be done carefully. Moreover, the immigration wave Europe has seen recently could not be assessed by any of the studies included in the review, making the application of the results to Germany and the current refugee crisis difficult.

Finally, cultural differences in explanatory models of psychotic experiences can render diagnostic procedures challenging, although substantial overlap in key symptoms has repeatedly been reported^24, 25^

## Concluding remarks

Recent studies, for example, from Germany^26^ show incidences of mental disorders in refugee populations, but due to small sample sizes and missing control groups conclusions on the incidence and risk evaluation of psychosis in refugees is limited.

The findings of this review suggest that trauma, social exclusion and discrimination may be critical factors in the multifactorial aetiology of psychotic disorders.^27, 28^ The outcomes are aggravated by the pre-migratory adversities many asylum seekers and refugees have experienced such as fear, violence, persecution and separation from families.^29^ Post-migration adversities in terms of social and economic disadvantage may also play a role in the development of psychosis contributing to symptoms and are detrimental to mental health.^29^ These include social isolation, discrimination, unemployment, poverty, family dysfunction, lack of stable housing and uncertainty about asylum applications.^29, 30, 31, 32^

Indeed, higher incidence rates for schizophrenia have been reported in people whose position in society is disadvantaged.^30^ The risk for developing psychosis is low in migrants living in the highest income areas and when the sending and receiving countries are similar, that is, Western country to Western country.^17, 29^ Living in poverty may have a genetic component.^33^ Also, it seems that negative effects on the mental health of refugees are attributable to the long periods taken to process asylum applications, further undermining the integration process.^29^ Hence, social and government policies and legislation can contribute to the alleviation of symptoms and risk factors.

Lastly, it should be mentioned that a notable disproportion in terms of gender can be observed among refugees: the majority of all asylum seekers are men and women account only for 30.8% of all refugees.^5^ On grounds of the social status and perception of women encountered in different cultural contexts and potential depreciation and subordination of women, a lower rate of utilisation of psychiatric care among female refugees due to their challenged status has to be taken into account.

At this point, the most needed intervention seems to be outreach programmes that target these susceptible patients and address the issues of language problems, cultural differences, poor health literacy and low rates of utilisation of psychiatric services.^22, 34, 35, 36, 37, 38^ Given the high susceptibility to psychotic disorders among refugees, preventive strategies that provide practical support for these patients should be developed and implemented. It is therefore crucial that clinicians and people working in a refugee setting are aware of early signs and symptoms and be sensitised to intercultural competence. Further studies are needed to determine effective therapies that meet the individual needs of this vulnerable population.

## Figures and Tables

**Table 1:** Main characteristics of the included studies

	*Title*	*Population*	*Age (mean age in years)*	*Gender (% of female participants) of the cases identified, person years*	*Follow-up*	*Diagnosis*	*Data analysis*
Hollander *et al.*^15^ (Sweden)	Refugee migration and risk of schizophrenia and other non-affective psychoses: cohort study of 1.3 million people in Sweden	Refugees, *n*=24 123 Non-refugee migrants, *n*=132 663 Swedish-born population, *n*=1 191 004	Born after 1984: 14 years and older	Refugees: 44.2% Non-refugee migrants: 50.8% Swedish-born population: 48.6%	8.9 Million person years First recorded diagnosis between 1 January 1998 and 31 December 2011	Diagnosis of non-affective psychotic disorder (ICD-10 F20–29)	Cox proportional hazard models, Lexis expansion for stratifying the participants according to the age at risk, adjustment for age at risk, sex, and their interaction, adjustment for disposable income and population density, sensitivity analyses, likelihood ratio tests
Anderson *et al.*^16^ (Ontario, Canada)	Incidence of psychotic disorders among first-generation immigrants and refugees in Ontario	Refugees, *n*=95 148 Immigrants, *n*=323 285 General population, *n*=3 866 261	14–40 Years of age as of 1 April 1999: Refugees: 29.7 Immigrants: 29.0 General population: 27.9	Refugees: 40.1% Immigrants: 51.5% General population: 49.7%	10 Years	Primary discharge diagnosis of schizophrenia or schizoaffective disorder from a general hospital bed (ICD-10 F20 or F25), psychiatric hospital bed (DSM-IV code 295.x) or billing claims or emergency department visits (ICD-10 F20 or F25)	Poisson regression adjusting for age and sex, Poisson regression with scaled deviance to estimate the independent effects of sex, age at migration, length of stay in Canada, urban residence, refugee status and income quintile
Norredam *et al.*^17^ (Denmark)	Risk of mental disorders in refugees and native Danes: a register-based retrospective cohort study	Refugees, *n*=29 139 Native Danes, *n*=116 556	18 Years and older: Refugees: 32.9 Native Danes: 32.9	Refugees: 44.4% Native Danes: 44.4%	Refugees: 8.0 years Native Danes: 8.1 years	First-time psychiatric contact, diagnosis of psychotic disorder (ICD-10 F20–29)	Poisson regression model including sex, age and region of origin

**Table 2:** Main results of the included studies

	*Refugees compared to control group(s)*	*Gender*	*Middle East as region of origin*
Hollander *et al.*^15^	Incidence rate per 100 000 person years: Refugees: 126.4 (103.1; 154.8) Migrants: 80.4 (72.7; 88.9) Swedish-born population: 38.5 (37.2; 39.9) Swedish-born as reference (hazard ratios): 3.61 (2.87; 4.53) after adjustment for age at risk, sex, and their interaction (model 1) 2.90 (2.31; 3.64) after adjustment for disposable income and population density (model 2) Non-refugee migrant as reference (hazard ratios): 1.58 (1.26; 1.99) (model 1) 1.66 (1.32; 2.09) (model 2)	Swedish-born as reference (hazard ratios): Men: 4.28 (3.28; 5.58) (model 1); 3.49 (2.67; 4.55) (model 2) Women: 2.65 (1.80; 3.92) (model 1); 2.07 (1.40; 3.06) (model 2) Non-refugee migrant as reference (hazard ratios) Men: 1.64 (1.25; 2.15) (model 1); 1.74 (1.32; 2.28) (model 2) Women: 1.39 (0.92; 2.10) (model 1); 1.43 (0.95; 2.16) (model 2)	Including N Africa CIR: 112.8 (82.7; 153.8), hazard ratio: 1.56 (1.08; 2.23) (model 2) (Swedish-born: CIR = 38.5 (37.2; 39.9); Middle Eastern non-refugee migrants: CIR = 70.9 (59.4; 84.6)) Men: CIR = 143.5 (100.3; 205.2), hazard ratio: 1.55 (1.01; 2.36) (model 2) (Swedish-born: CIR = 41.2 (39.4; 43.2); Middle Eastern non-refugees: CIR = 94.4 (75.9; 117.4)) Reference group for hazard ratios: non-refugees from same region
Anderson *et al.*^16^	Incidence rate per 100 000 person years: Refugees: 72.8 Migrants: 51.7 General population: 55.6 IRR: Refugees: 1.24 (0.86; 1.81) Migrants: 0.91 (0.71; 1.16, statistically not significant) Refugees compared to non-refugees, IRR: 1.27 (1.04; 1.56)	Male migrants compared with female migrants (refugee and non-refugee status), IRR: 1.45 (1.33; 1.58)	West Central Asia and Middle East Incidence rate per 100 000 person years: 57.2 (general population: 55.6); migrants, IRR: 0.75 (0.49; 1.15) (overall IRR among immigrants: 0.91 (0.71; 1.16)); refugees, IRR: 1.28 (0.91; 1.80) (overall IRR among refugees: 1.24 (0.86; 1.81))
Norredam *et al.*^17^	Psychotic disorders among refugees versus native population, RR: 2.03 (1.72; 2.40)	First-time psychiatric contact among refugees Women, RR: 1.49 (1.29; 1.72) Men, RR: 2.02 (1.75; 2.34)	First-time psychiatric contact Middle East including N Africa: women, RR: 1.60 (1.18; 2.19); men, RR: 2.76 (2.24; 3.41) Iraq: women, RR: 1.95 (1.47; 2.59); men, RR: 3.31 (2.85; 3.83) (reference group: native Danes, all refugees: RR = 1.49 (1.29; 1.72)) psychotic disorders, both sex: Iraq, RR: 2.30 (1.72; 3.08); Middle East including N Africa, RR: 3.10 (2.28; 4.21) (reference group: native Danes, all refugees: RR =2.03 (1.72; 2.40))

Abbreviations: CIR, crude incidence rate per 100 000 person years at risk; IRR, incidence rate ratio; RR, rate ratio.

95% confidence intervals are in parentheses. Model 1 refers to adjustment for age at risk, sex, and their interaction. Model 2 refers to additional adjustment for disposable income and population density.
